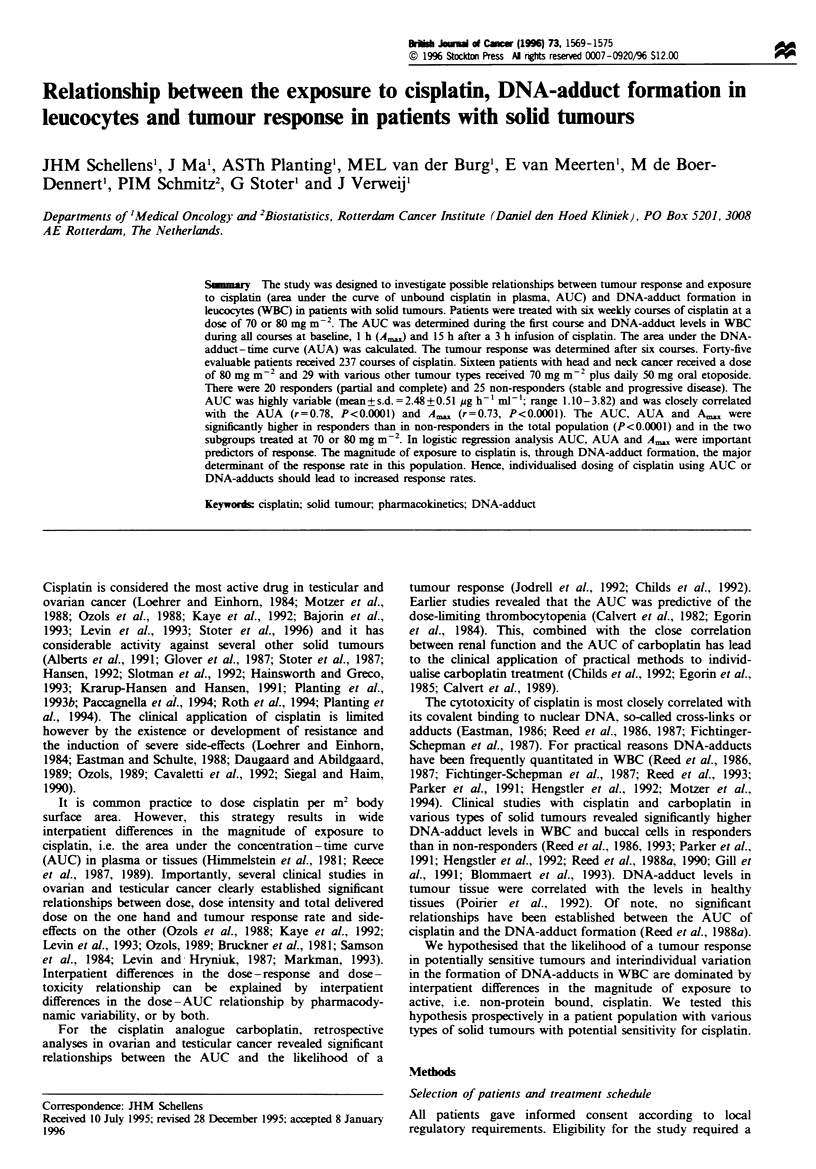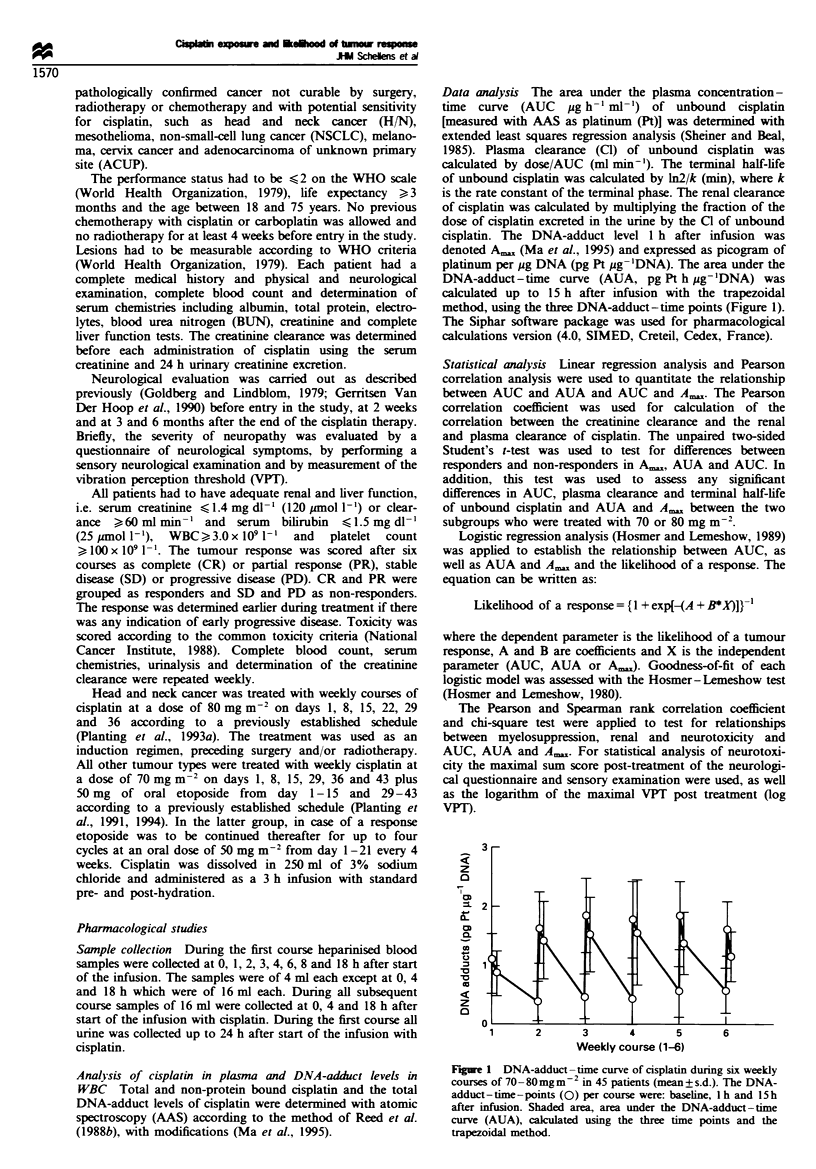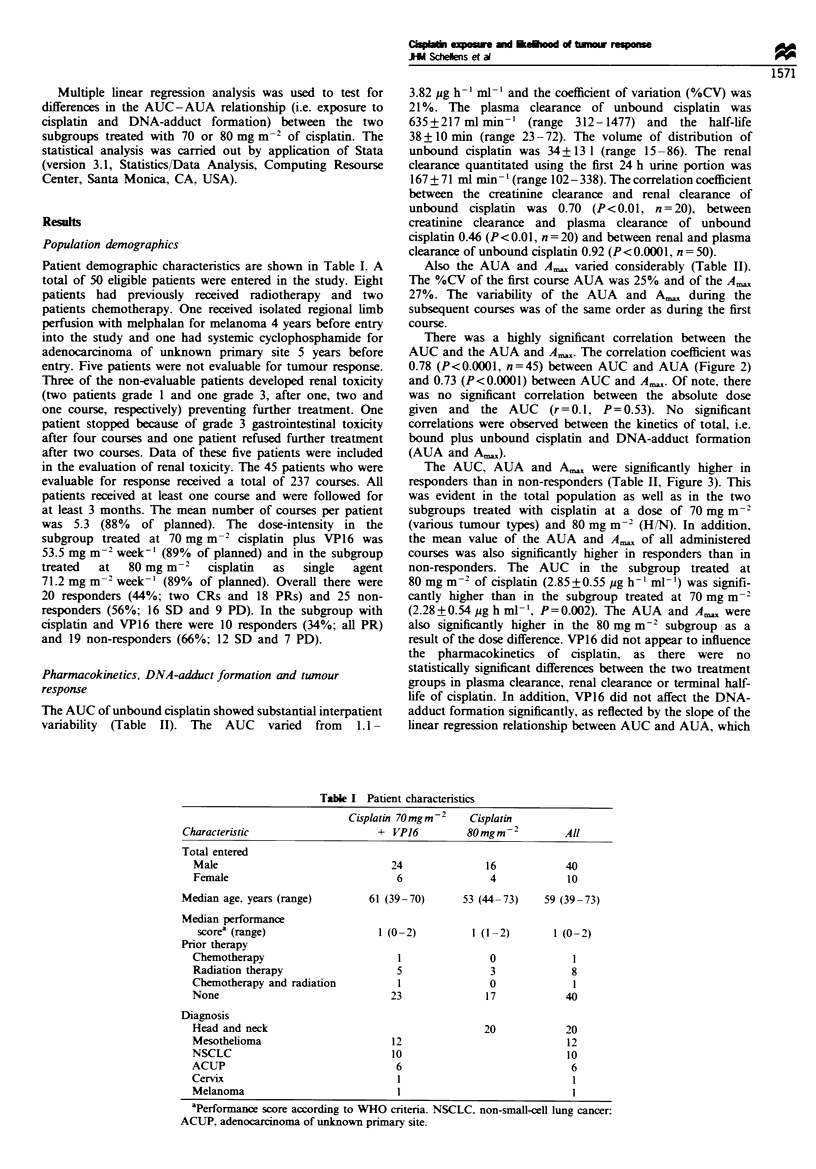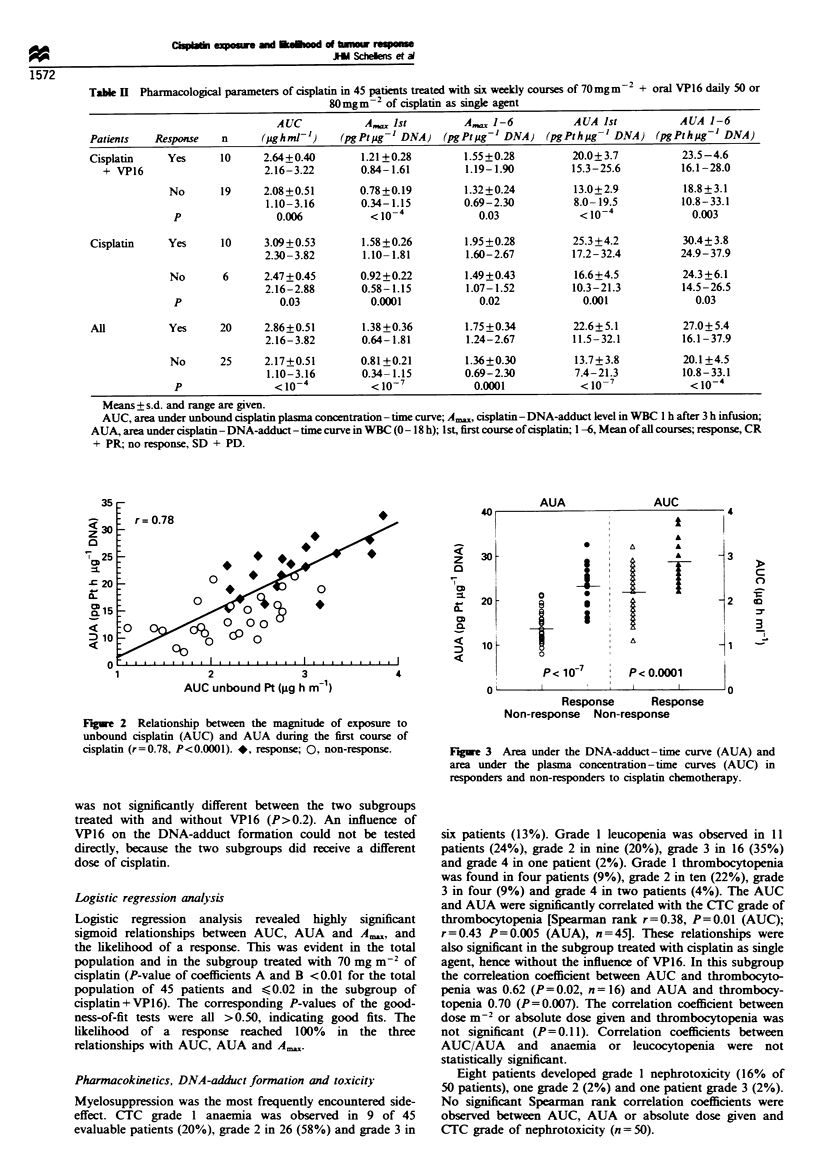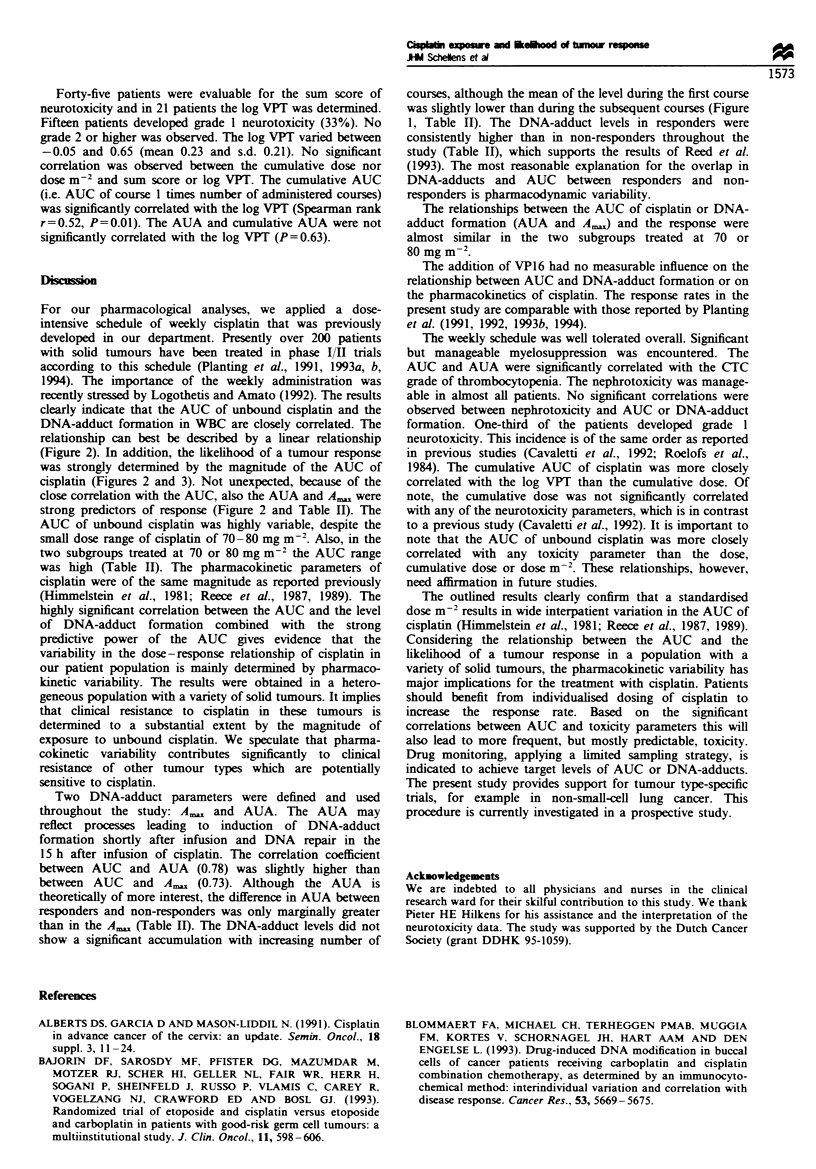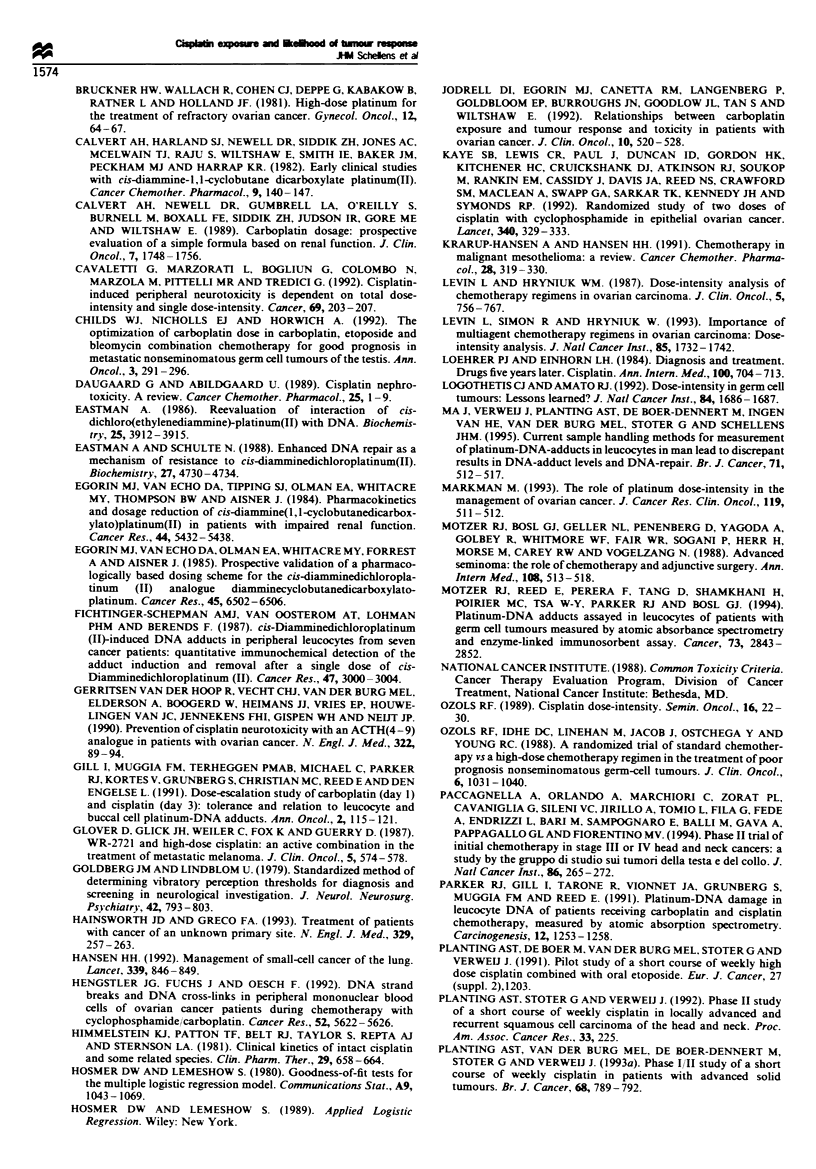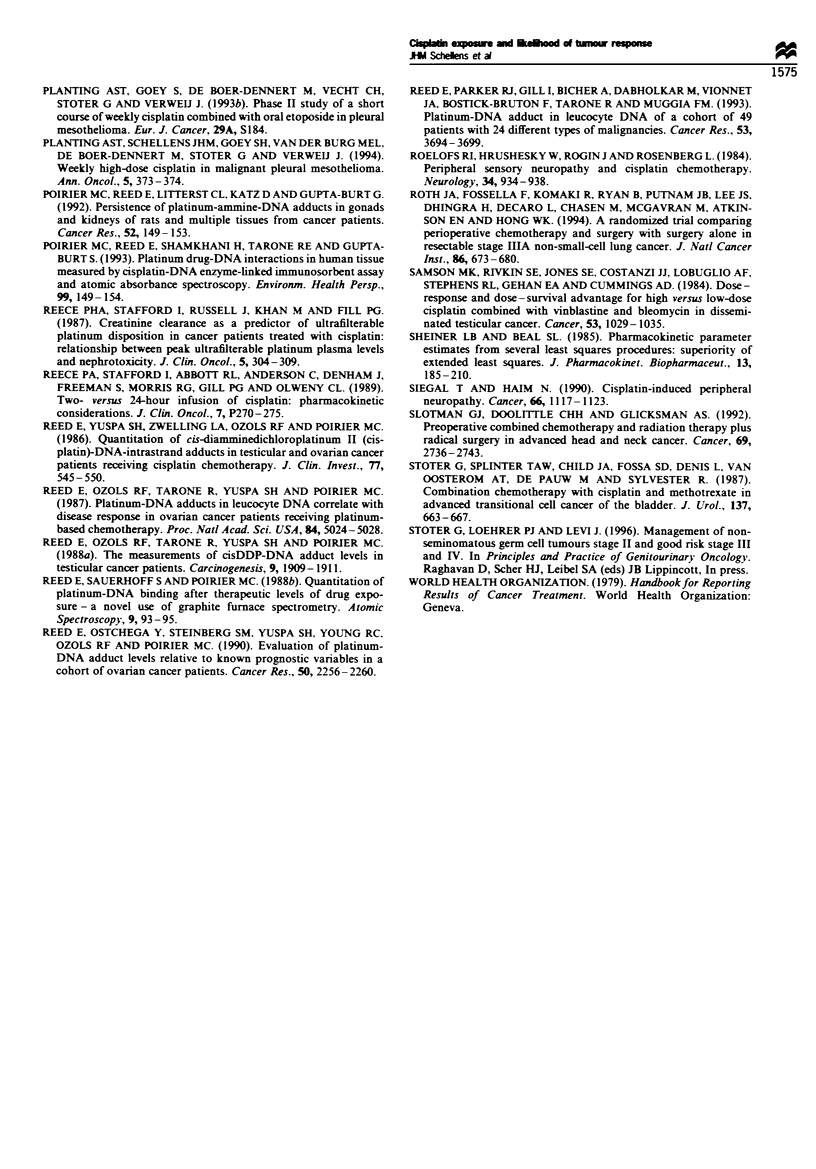# Relationship between the exposure to cisplatin, DNA-adduct formation in leucocytes and tumour response in patients with solid tumours.

**DOI:** 10.1038/bjc.1996.296

**Published:** 1996-06

**Authors:** J. H. Schellens, J. Ma, A. S. Planting, M. E. van der Burg, E. van Meerten, M. de Boer-Dennert, P. I. Schmitz, G. Stoter, J. Verweij

**Affiliations:** Department of Medical Oncology, Rotterdam Cancer Institute (Daniel den Hoed Kliniek), The Netherlands.

## Abstract

The study was designed to investigate possible relationships between tumour response and exposure to cisplatin (area under the curve of unbound cisplatin in plasma, AUC) and DNA-adduct formation in leucocytes (WBC) in patients with solid tumours. Patients were treated with six weekly courses of cisplatin at a dose of 70 or 80 mg m-2. The AUC was determined during the first course and DNA-adduct levels in WBC during all courses at baseline, 1 h (A(max)) and 15 h after a 3 h infusion of cisplatin. The area under the DNA-adduct-time curve (AUA) was calculated. The tumour response was determined after six courses. Forty-five evaluable patients received 237 courses of cisplatin. Sixteen patients with head and neck cancer received a dose of 80 mg m-2 and 29 with various other tumour types received 70 mg m-2 plus daily 50 mg oral etoposide. There were 20 responders (partial and complete) and 25 non-responders (stable and progressive disease). The AUC was highly variable (mean +/- s.d. = 2.48 +/- 0.51 micrograms h-1 ml-1; range 1.10-3.82) and was closely correlated with the AUA (r = 0.78, P < 0.0001) and A(max) (r = 0.73, P < 0.0001). The AUC, AUA and A(max) were significantly higher in responders than in non-responders in the total population (P < 0.0001) and in the two subgroups treated at 70 or 80 mg m-2. In logistic regression analysis AUC, AUA and A(max) were important predictors of response. The magnitude of exposure to cisplatin is, through DNA-adduct formation, the major determinant of the response rate in this population. Hence, individualised dosing of cisplatin using AUC or DNA-adducts should lead to increased response rates.


British Journal of Cancer (1996) 73, 1569-1575

© 1996 Stockton Press All rights reserved 0007-0920/96 $12.00

### Relationship between the exposure to cisplatin, DNA-adduct formation in leucocytes and tumour response in patients with solid tumours

JHM Schellens[1], J Ma[1], ASTh Planting[1], MEL van der Burg[1], E van Meerten[1], M de Boer-Dennert[1], PIM Schmitz[2], G Stoter[1] and J Verweij[1]

*Departments of [1]Medical Oncology and [2]Biostatistics, Rotterdam Cancer Institute (Daniel den Hoed Kliniek), PO Box 5201, 3008 AE Rotterdam, The Netherlands.*

**Summary** The study was designed to investigate possible relationships between tumour response and exposure to cisplatin (area under the curve of unbound cisplatin in plasma, AUC) and DNA-adduct formation in leucocytes (WBC) in patients with solid tumours. Patients were treated with six weekly courses of cisplatin at a dose of 70 or 80 mg $m^{-2}$. The AUC was determined during the first course and DNA-adduct levels in WBC during all courses at baseline, 1 h ($A_{max}$) and 15 h after a 3 h infusion of cisplatin. The area under the DNA-adduct-time curve (AUA) was calculated. The tumour response was determined after six courses. Forty-five evaluable patients received 237 courses of cisplatin. Sixteen patients with head and neck cancer received a dose of 80 mg $m^{-2}$ and 29 with various other tumour types received 70 mg $m^{-2}$ plus daily 50 mg oral etoposide. There were 20 responders (partial and complete) and 25 non-responders (stable and progressive disease). The AUC was highly variable (mean±s.d. = 2.48±0.51 $\mu g\ h^{-1}\ ml^{-1}$; range 1.10-3.82) and was closely correlated with the AUA ($r$=0.78, $P$<0.0001) and $A_{max}$ ($r$=0.73, $P$<0.0001). The AUC, AUA and $A_{max}$ were significantly higher in responders than in non-responders in the total population ($P$<0.0001) and in the two subgroups treated at 70 or 80 mg $m^{-2}$. In logistic regression analysis AUC, AUA and $A_{max}$ were important predictors of response. The magnitude of exposure to cisplatin is, through DNA-adduct formation, the major determinant of the response rate in this population. Hence, individualised dosing of cisplatin using AUC or DNA-adducts should lead to increased response rates.

**Keywords:** cisplatin; solid tumour; pharmacokinetics; DNA-adduct

Cisplatin is considered the most active drug in testicular and ovarian cancer (Loehrer and Einhorn, 1984; Motzer *et al.*, 1988; Ozols *et al.*, 1988; Kaye *et al.*, 1992; Bajorin *et al.*, 1993; Levin *et al.*, 1993; Stoter *et al.*, 1996) and it has considerable activity against several other solid tumours (Alberts *et al.*, 1991; Glover *et al.*, 1987; Stoter *et al.*, 1987; Hansen, 1992; Slotman *et al.*, 1992; Hainsworth and Greco, 1993; Krarup-Hansen and Hansen, 1991; Planting *et al.*, 1993*b*; Paccagnella *et al.*, 1994; Roth *et al.*, 1994; Planting *et al.*, 1994). The clinical application of cisplatin is limited however by the existence or development of resistance and the induction of severe side-effects (Loehrer and Einhorn, 1984; Eastman and Schulte, 1988; Daugaard and Abildgaard, 1989; Ozols, 1989; Cavaletti *et al.*, 1992; Siegal and Haim, 1990).

It is common practice to dose cisplatin per $m^2$ body surface area. However, this strategy results in wide interpatient differences in the magnitude of exposure to cisplatin, i.e. the area under the concentration-time curve (AUC) in plasma or tissues (Himmelstein *et al.*, 1981; Reece *et al.*, 1987, 1989). Importantly, several clinical studies in ovarian and testicular cancer clearly established significant relationships between dose, dose intensity and total delivered dose on the one hand and tumour response rate and side-effects on the other (Ozols *et al.*, 1988; Kaye *et al.*, 1992; Levin *et al.*, 1993; Ozols, 1989; Bruckner *et al.*, 1981; Samson *et al.*, 1984; Levin and Hryniuk, 1987; Markman, 1993). Interpatient differences in the dose-response and dose-toxicity relationship can be explained by interpatient differences in the dose-AUC relationship by pharmacodynamic variability, or by both.

For the cisplatin analogue carboplatin, retrospective analyses in ovarian and testicular cancer revealed significant relationships between the AUC and the likelihood of a tumour response (Jodrell *et al.*, 1992; Childs *et al.*, 1992). Earlier studies revealed that the AUC was predictive of the dose-limiting thrombocytopenia (Calvert *et al.*, 1982; Egorin *et al.*, 1984). This, combined with the close correlation between renal function and the AUC of carboplatin has lead to the clinical application of practical methods to individualise carboplatin treatment (Childs *et al.*, 1992; Egorin *et al.*, 1985; Calvert *et al.*, 1989).

The cytotoxicity of cisplatin is most closely correlated with its covalent binding to nuclear DNA, so-called cross-links or adducts (Eastman, 1986; Reed *et al.*, 1986, 1987; Fichtinger-Schepman *et al.*, 1987). For practical reasons DNA-adducts have been frequently quantitated in WBC (Reed *et al.*, 1986, 1987; Fichtinger-Schepman *et al.*, 1987; Reed *et al.*, 1993; Parker *et al.*, 1991; Hengstler *et al.*, 1992; Motzer *et al.*, 1994). Clinical studies with cisplatin and carboplatin in various types of solid tumours revealed significantly higher DNA-adduct levels in WBC and buccal cells in responders than in non-responders (Reed *et al.*, 1986, 1993; Parker *et al.*, 1991; Hengstler *et al.*, 1992; Reed *et al.*, 1988*a*, 1990; Gill *et al.*, 1991; Blommaert *et al.*, 1993). DNA-adduct levels in tumour tissue were correlated with the levels in healthy tissues (Poirier *et al.*, 1992). Of note, no significant relationships have been established between the AUC of cisplatin and the DNA-adduct formation (Reed *et al.*, 1988*a*).

We hypothesised that the likelihood of a tumour response in potentially sensitive tumours and interindividual variation in the formation of DNA-adducts in WBC are dominated by interpatient differences in the magnitude of exposure to active, i.e. non-protein bound, cisplatin. We tested this hypothesis prospectively in a patient population with various types of solid tumours with potential sensitivity for cisplatin.

### Methods

### *Selection of patients and treatment schedule*

All patients gave informed consent according to local regulatory requirements. Eligibility for the study required a

Correspondence: JHM Schellens
Received 10 July 1995; revised 28 December 1995; accepted 8 January 1996

pathologically confirmed cancer not curable by surgery, radiotherapy or chemotherapy and with potential sensitivity for cisplatin, such as head and neck cancer (H/N), mesothelioma, non-small-cell lung cancer (NSCLC), melanoma, cervix cancer and adenocarcinoma of unknown primary site (ACUP).

The performance status had to be $\leqslant 2$ on the WHO scale (World Health Organization, 1979), life expectancy $\geqslant 3$ months and the age between 18 and 75 years. No previous chemotherapy with cisplatin or carboplatin was allowed and no radiotherapy for at least 4 weeks before entry in the study. Lesions had to be measurable according to WHO criteria (World Health Organization, 1979). Each patient had a complete medical history and physical and neurological examination, complete blood count and determination of serum chemistries including albumin, total protein, electrolytes, blood urea nitrogen (BUN), creatinine and complete liver function tests. The creatinine clearance was determined before each administration of cisplatin using the serum creatinine and 24 h urinary creatinine excretion.

Neurological evaluation was carried out as described previously (Goldberg and Lindblom, 1979; Gerritsen Van Der Hoop *et al*., 1990) before entry in the study, at 2 weeks and at 3 and 6 months after the end of the cisplatin therapy. Briefly, the severity of neuropathy was evaluated by a questionnaire of neurological symptoms, by performing a sensory neurological examination and by measurement of the vibration perception threshold (VPT).

All patients had to have adequate renal and liver function, i.e. serum creatinine $\leqslant 1.4$ mg dl$^{-1}$ (120 $\mu$mol l$^{-1}$) or clearance $\geqslant 60$ ml min$^{-1}$ and serum bilirubin $\leqslant 1.5$ mg dl$^{-1}$ (25 $\mu$mol l$^{-1}$), WBC $\geqslant 3.0 \times 10^9$ l$^{-1}$ and platelet count $\geqslant 100 \times 10^9$ l$^{-1}$. The tumour response was scored after six courses as complete (CR) or partial response (PR), stable disease (SD) or progressive disease (PD). CR and PR were grouped as responders and SD and PD as non-responders. The response was determined earlier during treatment if there was any indication of early progressive disease. Toxicity was scored according to the common toxicity criteria (National Cancer Institute, 1988). Complete blood count, serum chemistries, urinalysis and determination of the creatinine clearance were repeated weekly.

Head and neck cancer was treated with weekly courses of cisplatin at a dose of 80 mg m$^{-2}$ on days 1, 8, 15, 22, 29 and 36 according to a previously established schedule (Planting *et al*., 1993*a*). The treatment was used as an induction regimen, preceding surgery and/or radiotherapy. All other tumour types were treated with weekly cisplatin at a dose of 70 mg m$^{-2}$ on days 1, 8, 15, 29, 36 and 43 plus 50 mg of oral etoposide from day 1–15 and 29–43 according to a previously established schedule (Planting *et al*., 1991, 1994). In the latter group, in case of a response etoposide was to be continued thereafter for up to four cycles at an oral dose of 50 mg m$^{-2}$ from day 1–21 every 4 weeks. Cisplatin was dissolved in 250 ml of 3% sodium chloride and administered as a 3 h infusion with standard pre- and post-hydration.

### *Pharmacological studies*

*Sample collection* During the first course heparinised blood samples were collected at 0, 1, 2, 3, 4, 6, 8 and 18 h after start of the infusion. The samples were of 4 ml each except at 0, 4 and 18 h which were of 16 ml each. During all subsequent course samples of 16 ml were collected at 0, 4 and 18 h after start of the infusion with cisplatin. During the first course all urine was collected up to 24 h after start of the infusion with cisplatin.

*Analysis of cisplatin in plasma and DNA-adduct levels in WBC* Total and non-protein bound cisplatin and the total DNA-adduct levels of cisplatin were determined with atomic spectroscopy (AAS) according to the method of Reed *et al*. (1988*b*), with modifications (Ma *et al*., 1995).

*Data analysis* The area under the plasma concentration–time curve (AUC $\mu$g h$^{-1}$ ml$^{-1}$) of unbound cisplatin [measured with AAS as platinum (Pt)] was determined with extended least squares regression analysis (Sheiner and Beal, 1985). Plasma clearance (Cl) of unbound cisplatin was calculated by dose/AUC (ml min$^{-1}$). The terminal half-life of unbound cisplatin was calculated by $\ln 2/k$ (min), where $k$ is the rate constant of the terminal phase. The renal clearance of cisplatin was calculated by multiplying the fraction of the dose of cisplatin excreted in the urine by the Cl of unbound cisplatin. The DNA-adduct level 1 h after infusion was denoted $A_{max}$ (Ma *et al*., 1995) and expressed as picogram of platinum per $\mu$g DNA (pg Pt $\mu$g$^{-1}$DNA). The area under the DNA-adduct–time curve (AUA, pg Pt h $\mu$g$^{-1}$DNA) was calculated up to 15 h after infusion with the trapezoidal method, using the three DNA-adduct–time points (Figure 1). The Siphar software package was used for pharmacological calculations version (4.0, SIMED, Creteil, Cedex, France).

*Statistical analysis* Linear regression analysis and Pearson correlation analysis were used to quantitate the relationship between AUC and AUA and AUC and $A_{max}$. The Pearson correlation coefficient was used for calculation of the correlation between the creatinine clearance and the renal and plasma clearance of cisplatin. The unpaired two-sided Student's $t$-test was used to test for differences between responders and non-responders in $A_{max}$, AUA and AUC. In addition, this test was used to assess any significant differences in AUC, plasma clearance and terminal half-life of unbound cisplatin and AUA and $A_{max}$ between the two subgroups who were treated with 70 or 80 mg m$^{-2}$.

Logistic regression analysis (Hosmer and Lemeshow, 1989) was applied to establish the relationship between AUC, as well as AUA and $A_{max}$ and the likelihood of a response. The equation can be written as:

$$\text{Likelihood of a response} = \{1 + \exp[-(A + B^{*}X)]\}^{-1}$$

where the dependent parameter is the likelihood of a tumour response, A and B are coefficients and X is the independent parameter (AUC, AUA or $A_{max}$). Goodness-of-fit of each logistic model was assessed with the Hosmer–Lemeshow test (Hosmer and Lemeshow, 1980).

The Pearson and Spearman rank correlation coefficient and chi-square test were applied to test for relationships between myelosuppression, renal and neurotoxicity and AUC, AUA and $A_{max}$. For statistical analysis of neurotoxicity the maximal sum score post-treatment of the neurological questionnaire and sensory examination were used, as well as the logarithm of the maximal VPT post treatment (log VPT).

**Figure 1** DNA-adduct–time curve of cisplatin during six weekly courses of 70–80 mg m$^{-2}$ in 45 patients (mean ± s.d.). The DNA-adduct–time-points (○) per course were: baseline, 1 h and 15 h after infusion. Shaded area, area under the DNA-adduct–time curve (AUA), calculated using the three time points and the trapezoidal method.

Multiple linear regression analysis was used to test for differences in the AUC–AUA relationship (i.e. exposure to cisplatin and DNA-adduct formation) between the two subgroups treated with 70 or 80 mg m$^{-2}$ of cisplatin. The statistical analysis was carried out by application of Stata (version 3.1, Statistics/Data Analysis, Computing Resourse Center, Santa Monica, CA, USA).

### Results

### *Population demographics*

Patient demographic characteristics are shown in Table I. A total of 50 eligible patients were entered in the study. Eight patients had previously received radiotherapy and two patients chemotherapy. One received isolated regional limb perfusion with melphalan for melanoma 4 years before entry into the study and one had systemic cyclophosphamide for adenocarcinoma of unknown primary site 5 years before entry. Five patients were not evaluable for tumour response. Three of the non-evaluable patients developed renal toxicity (two patients grade 1 and one grade 3, after one, two and one course, respectively) preventing further treatment. One patient stopped because of grade 3 gastrointestinal toxicity after four courses and one patient refused further treatment after two courses. Data of these five patients were included in the evaluation of renal toxicity. The 45 patients who were evaluable for response received a total of 237 courses. All patients received at least one course and were followed for at least 3 months. The mean number of courses per patient was 5.3 (88% of planned). The dose-intensity in the subgroup treated at 70 mg m$^{-2}$ cisplatin plus VP16 was 53.5 mg m$^{-2}$ week$^{-1}$ (89% of planned) and in the subgroup treated at 80 mg m$^{-2}$ cisplatin as single agent 71.2 mg m$^{-2}$ week$^{-1}$ (89% of planned). Overall there were 20 responders (44%; two CRs and 18 PRs) and 25 non-responders (56%; 16 SD and 9 PD). In the subgroup with cisplatin and VP16 there were 10 responders (34%; all PR) and 19 non-responders (66%; 12 SD and 7 PD).

### *Pharmacokinetics, DNA-adduct formation and tumour response*

The AUC of unbound cisplatin showed substantial interpatient variability (Table II). The AUC varied from 1.1–3.82 $\mu$g h$^{-1}$ ml$^{-1}$ and the coefficient of variation (%CV) was 21%. The plasma clearance of unbound cisplatin was 635±217 ml min$^{-1}$ (range 312–1477) and the half-life 38±10 min (range 23–72). The volume of distribution of unbound cisplatin was 34±13 l (range 15–86). The renal clearance quantitated using the first 24 h urine portion was 167±71 ml min$^{-1}$ (range 102–338). The correlation coefficient between the creatinine clearance and renal clearance of unbound cisplatin was 0.70 ($P<0.01$, $n=20$), between creatinine clearance and plasma clearance of unbound cisplatin 0.46 ($P<0.01$, $n=20$) and between renal and plasma clearance of unbound cisplatin 0.92 ($P<0.0001$, $n=50$).

Also the AUA and $A_{max}$ varied considerably (Table II). The %CV of the first course AUA was 25% and of the $A_{max}$ 27%. The variability of the AUA and $A_{max}$ during the subsequent courses was of the same order as during the first course.

There was a highly significant correlation between the AUC and the AUA and $A_{max}$. The correlation coefficient was 0.78 ($P<0.0001$, $n=45$) between AUC and AUA (Figure 2) and 0.73 ($P<0.0001$) between AUC and $A_{max}$. Of note, there was no significant correlation between the absolute dose given and the AUC ($r=0.1$, $P=0.53$). No significant correlations were observed between the kinetics of total, i.e. bound plus unbound cisplatin and DNA-adduct formation (AUA and $A_{max}$).

The AUC, AUA and $A_{max}$ were significantly higher in responders than in non-responders (Table II, Figure 3). This was evident in the total population as well as in the two subgroups treated with cisplatin at a dose of 70 mg m$^{-2}$ (various tumour types) and 80 mg m$^{-2}$ (H/N). In addition, the mean value of the AUA and $A_{max}$ of all administered courses was also significantly higher in responders than in non-responders. The AUC in the subgroup treated at 80 mg m$^{-2}$ of cisplatin (2.85±0.55 $\mu$g h$^{-1}$ ml$^{-1}$) was significantly higher than in the subgroup treated at 70 mg m$^{-2}$ (2.28±0.54 $\mu$g h ml$^{-1}$, $P=0.002$). The AUA and $A_{max}$ were also significantly higher in the 80 mg m$^{-2}$ subgroup as a result of the dose difference. VP16 did not appear to influence the pharmacokinetics of cisplatin, as there were no statistically significant differences between the two treatment groups in plasma clearance, renal clearance or terminal half-life of cisplatin. In addition, VP16 did not affect the DNA-adduct formation significantly, as reflected by the slope of the linear regression relationship between AUC and AUA, which

**Table I** Patient characteristics

| *Characteristic* | *Cisplatin 70 mg m$^{-2}$ + VP16* | *Cisplatin 80 mg m$^{-2}$* | *All* |
|---|---|---|---|
| Total entered | | | |
| Male | 24 | 16 | 40 |
| Female | 6 | 4 | 10 |
| Median age, years (range) | 61 (39–70) | 53 (44–73) | 59 (39–73) |
| Median performance score[a] (range) | 1 (0–2) | 1 (1–2) | 1 (0–2) |
| Prior therapy | | | |
| Chemotherapy | 1 | 0 | 1 |
| Radiation therapy | 5 | 3 | 8 |
| Chemotherapy and radiation | 1 | 0 | 1 |
| None | 23 | 17 | 40 |
| Diagnosis | | | |
| Head and neck | | 20 | 20 |
| Mesothelioma | 12 | | 12 |
| NSCLC | 10 | | 10 |
| ACUP | 6 | | 6 |
| Cervix | 1 | | 1 |
| Melanoma | 1 | | 1 |

[a]Performance score according to WHO criteria. NSCLC, non-small-cell lung cancer; ACUP, adenocarcinoma of unknown primary site.

**Table II** Pharmacological parameters of cisplatin in 45 patients treated with six weekly courses of $70\,mg\,m^{-2}$ + oral VP16 daily 50 or $80\,mg\,m^{-2}$ of cisplatin as single agent

| *Patients* | *Response* | n | *AUC* ($\mu g\,h\,ml^{-1}$) | $A_{max}$ *1st* ($pg\,Pt\,\mu g^{-1}$ *DNA*) | $A_{max}$ *1–6* ($pg\,Pt\,\mu g^{-1}$ *DNA*) | *AUA 1st* ($pg\,Pt\,h\,\mu g^{-1}$ *DNA*) | *AUA 1–6* ($pg\,Pt\,h\,\mu g^{-1}$ *DNA*) |
|---|---|---|---|---|---|---|---|
| Cisplatin + VP16 | Yes | 10 | 2.64±0.40 | 1.21±0.28 | 1.55±0.28 | 20.0±3.7 | 23.5–4.6 |
| | | | 2.16–3.22 | 0.84–1.61 | 1.19–1.90 | 15.3–25.6 | 16.1–28.0 |
| | No | 19 | 2.08±0.51 | 0.78±0.19 | 1.32±0.24 | 13.0±2.9 | 18.8±3.1 |
| | | | 1.10–3.16 | 0.34–1.15 | 0.69–2.30 | 8.0–19.5 | 10.8–33.1 |
| | *P* | | 0.006 | $<10^{-4}$ | 0.03 | $<10^{-4}$ | 0.003 |
| Cisplatin | Yes | 10 | 3.09±0.53 | 1.58±0.26 | 1.95±0.28 | 25.3±4.2 | 30.4±3.8 |
| | | | 2.30–3.82 | 1.10–1.81 | 1.60–2.67 | 17.2–32.4 | 24.9–37.9 |
| | No | 6 | 2.47±0.45 | 0.92±0.22 | 1.49±0.43 | 16.6±4.5 | 24.3±6.1 |
| | | | 2.16–2.88 | 0.58–1.15 | 1.07–1.52 | 10.3–21.3 | 14.5–26.5 |
| | *P* | | 0.03 | 0.0001 | 0.02 | 0.001 | 0.03 |
| All | Yes | 20 | 2.86±0.51 | 1.38±0.36 | 1.75±0.34 | 22.6±5.1 | 27.0±5.4 |
| | | | 2.16–3.82 | 0.64–1.81 | 1.24–2.67 | 11.5–32.1 | 16.1–37.9 |
| | No | 25 | 2.17±0.51 | 0.81±0.21 | 1.36±0.30 | 13.7±3.8 | 20.1±4.5 |
| | | | 1.10–3.16 | 0.34–1.15 | 0.69–2.30 | 7.4–21.3 | 10.8–33.1 |
| | *P* | | $<10^{-4}$ | $<10^{-7}$ | 0.0001 | $<10^{-7}$ | $<10^{-4}$ |

Means ± s.d. and range are given.
AUC, area under unbound cisplatin plasma concentration–time curve; $A_{max}$, cisplatin–DNA-adduct level in WBC 1 h after 3 h infusion; AUA, area under cisplatin–DNA-adduct–time curve in WBC (0–18 h); 1st, first course of cisplatin; 1–6, Mean of all courses; response, CR + PR; no response, SD + PD.

**Figure 2** Relationship between the magnitude of exposure to unbound cisplatin (AUC) and AUA during the first course of cisplatin ($r=0.78$, $P<0.0001$). ◆, response; ○, non-response.

**Figure 3** Area under the DNA-adduct–time curve (AUA) and area under the plasma concentration–time curves (AUC) in responders and non-responders to cisplatin chemotherapy.

was not significantly different between the two subgroups treated with and without VP16 ($P>0.2$). An influence of VP16 on the DNA-adduct formation could not be tested directly, because the two subgroups did receive a different dose of cisplatin.

*Logistic regression analysis*

Logistic regression analysis revealed highly significant sigmoid relationships between AUC, AUA and $A_{max}$, and the likelihood of a response. This was evident in the total population and in the subgroup treated with 70 mg $m^{-2}$ of cisplatin ($P$-value of coefficients A and B $<0.01$ for the total population of 45 patients and $\leqslant 0.02$ in the subgroup of cisplatin + VP16). The corresponding $P$-values of the goodness-of-fit tests were all $>0.50$, indicating good fits. The likelihood of a response reached 100% in the three relationships with AUC, AUA and $A_{max}$.

*Pharmacokinetics, DNA-adduct formation and toxicity*

Myelosuppression was the most frequently encountered side-effect. CTC grade 1 anaemia was observed in 9 of 45 evaluable patients (20%), grade 2 in 26 (58%) and grade 3 in six patients (13%). Grade 1 leucopenia was observed in 11 patients (24%), grade 2 in nine (20%), grade 3 in 16 (35%) and grade 4 in one patient (2%). Grade 1 thrombocytopenia was found in four patients (9%), grade 2 in ten (22%), grade 3 in four (9%) and grade 4 in two patients (4%). The AUC and AUA were significantly correlated with the CTC grade of thrombocytopenia [Spearman rank $r=0.38$, $P=0.01$ (AUC); $r=0.43$ $P=0.005$ (AUA), $n=45$]. These relationships were also significant in the subgroup treated with cisplatin as single agent, hence without the influence of VP16. In this subgroup the correleation coefficient between AUC and thrombocytopenia was 0.62 ($P=0.02$, $n=16$) and AUA and thrombocytopenia 0.70 ($P=0.007$). The correlation coefficient between dose $m^{-2}$ or absolute dose given and thrombocytopenia was not significant ($P=0.11$). Correlation coefficients between AUC/AUA and anaemia or leucocytopenia were not statistically significant.

Eight patients developed grade 1 nephrotoxicity (16% of 50 patients), one grade 2 (2%) and one patient grade 3 (2%). No significant Spearman rank correlation coefficients were observed between AUC, AUA or absolute dose given and CTC grade of nephrotoxicity ($n=50$).

Forty-five patients were evaluable for the sum score of neurotoxicity and in 21 patients the log VPT was determined. Fifteen patients developed grade 1 neurotoxicity (33%). No grade 2 or higher was observed. The log VPT varied between −0.05 and 0.65 (mean 0.23 and s.d. 0.21). No significant correlation was observed between the cumulative dose nor dose $m^{-2}$ and sum score or log VPT. The cumulative AUC (i.e. AUC of course 1 times number of administered courses) was significantly correlated with the log VPT (Spearman rank $r=0.52$, $P=0.01$). The AUA and cumulative AUA were not significantly correlated with the log VPT ($P=0.63$).

### Discussion

For our pharmacological analyses, we applied a dose-intensive schedule of weekly cisplatin that was previously developed in our department. Presently over 200 patients with solid tumours have been treated in phase I/II trials according to this schedule (Planting *et al.*, 1991, 1993*a*, *b*, 1994). The importance of the weekly administration was recently stressed by Logothetis and Amato (1992). The results clearly indicate that the AUC of unbound cisplatin and the DNA-adduct formation in WBC are closely correlated. The relationship can best be described by a linear relationship (Figure 2). In addition, the likelihood of a tumour response was strongly determined by the magnitude of the AUC of cisplatin (Figures 2 and 3). Not unexpected, because of the close correlation with the AUC, also the AUA and $A_{max}$ were strong predictors of response (Figure 2 and Table II). The AUC of unbound cisplatin was highly variable, despite the small dose range of cisplatin of 70–80 mg $m^{-2}$. Also, in the two subgroups treated at 70 or 80 mg $m^{-2}$ the AUC range was high (Table II). The pharmacokinetic parameters of cisplatin were of the same magnitude as reported previously (Himmelstein *et al.*, 1981; Reece *et al.*, 1987, 1989). The highly significant correlation between the AUC and the level of DNA-adduct formation combined with the strong predictive power of the AUC gives evidence that the variability in the dose–response relationship of cisplatin in our patient population is mainly determined by pharmacokinetic variability. The results were obtained in a heterogeneous population with a variety of solid tumours. It implies that clinical resistance to cisplatin in these tumours is determined to a substantial extent by the magnitude of exposure to unbound cisplatin. We speculate that pharmacokinetic variability contributes significantly to clinical resistance of other tumour types which are potentially sensitive to cisplatin.

Two DNA-adduct parameters were defined and used throughout the study: $A_{max}$ and AUA. The AUA may reflect processes leading to induction of DNA-adduct formation shortly after infusion and DNA repair in the 15 h after infusion of cisplatin. The correlation coefficient between AUC and AUA (0.78) was slightly higher than between AUC and $A_{max}$ (0.73). Although the AUA is theoretically of more interest, the difference in AUA between responders and non-responders was only marginally greater than in the $A_{max}$ (Table II). The DNA-adduct levels did not show a significant accumulation with increasing number of courses, although the mean of the level during the first course was slightly lower than during the subsequent courses (Figure 1, Table II). The DNA-adduct levels in responders were consistently higher than in non-responders throughout the study (Table II), which supports the results of Reed *et al.* (1993). The most reasonable explanation for the overlap in DNA-adducts and AUC between responders and non-responders is pharmacodynamic variability.

The relationships between the AUC of cisplatin or DNA-adduct formation (AUA and $A_{max}$) and the response were almost similar in the two subgroups treated at 70 or 80 mg $m^{-2}$.

The addition of VP16 had no measurable influence on the relationship between AUC and DNA-adduct formation or on the pharmacokinetics of cisplatin. The response rates in the present study are comparable with those reported by Planting *et al.* (1991, 1992, 1993*b*, 1994).

The weekly schedule was well tolerated overall. Significant but manageable myelosuppression was encountered. The AUC and AUA were significantly correlated with the CTC grade of thrombocytopenia. The nephrotoxicity was manageable in almost all patients. No significant correlations were observed between nephrotoxicity and AUC or DNA-adduct formation. One-third of the patients developed grade 1 neurotoxicity. This incidence is of the same order as reported in previous studies (Cavaletti *et al.*, 1992; Roelofs *et al.*, 1984). The cumulative AUC of cisplatin was more closely correlated with the log VPT than the cumulative dose. Of note, the cumulative dose was not significantly correlated with any of the neurotoxicity parameters, which is in contrast to a previous study (Cavaletti *et al.*, 1992). It is important to note that the AUC of unbound cisplatin was more closely correlated with any toxicity parameter than the dose, cumulative dose or dose $m^{-2}$. These relationships, however, need affirmation in future studies.

The outlined results clearly confirm that a standardised dose $m^{-2}$ results in wide interpatient variation in the AUC of cisplatin (Himmelstein *et al.*, 1981; Reece *et al.*, 1987, 1989). Considering the relationship between the AUC and the likelihood of a tumour response in a population with a variety of solid tumours, the pharmacokinetic variability has major implications for the treatment with cisplatin. Patients should benefit from individualised dosing of cisplatin to increase the response rate. Based on the significant correlations between AUC and toxicity parameters this will also lead to more frequent, but mostly predictable, toxicity. Drug monitoring, applying a limited sampling strategy, is indicated to achieve target levels of AUC or DNA-adducts. The present study provides support for tumour type-specific trials, for example in non-small-cell lung cancer. This procedure is currently investigated in a prospective study.

**Acknowledgements**
We are indebted to all physicians and nurses in the clinical research ward for their skilful contribution to this study. We thank Pieter HE Hilkens for his assistance and the interpretation of the neurotoxicity data. The study was supported by the Dutch Cancer Society (grant DDHK 95-1059).

### References

ALBERTS DS, GARCIA D AND MASON-LIDDIL N. (1991). Cisplatin in advance cancer of the cervix: an update. *Semin. Oncol.*, **18** suppl. 3, 11–24.

BAJORIN DF, SAROSDY MF, PFISTER DG, MAZUMDAR M, MOTZER RJ, SCHER HI, GELLER NL, FAIR WR, HERR H, SOGANI P, SHEINFELD J, RUSSO P, VLAMIS C, CAREY R, VOGELZANG NJ, CRAWFORD ED AND BOSL GJ. (1993). Randomized trial of etoposide and cisplatin versus etoposide and carboplatin in patients with good-risk germ cell tumours: a multiinstitutional study. *J. Clin. Oncol.*, **11**, 598–606.

BLOMMAERT FA, MICHAEL CH, TERHEGGEN PMAB, MUGGIA FM, KORTES V, SCHORNAGEL JH, HART AAM AND DEN ENGELSE L. (1993). Drug-induced DNA modification in buccal cells of cancer patients receiving carboplatin and cisplatin combination chemotherapy, as determined by an immunocytochemical method: interindividual variation and correlation with disease response. *Cancer Res.*, **53**, 5669–5675.

BRUCKNER HW, WALLACH R, COHEN CJ, DEPPE G, KABAKOW B, RATNER L AND HOLLAND JF. (1981). High-dose platinum for the treatment of refractory ovarian cancer. *Gynecol. Oncol.*, **12**, 64-67.

CALVERT AH, HARLAND SJ, NEWELL DR, SIDDIK ZH, JONES AC, MCELWAIN TJ, RAJU S, WILTSHAW E, SMITH IE, BAKER JM, PECKHAM MJ AND HARRAP KR. (1982). Early clinical studies with *cis*-diammine-1,1-cyclobutane dicarboxylate platinum(II). *Cancer Chemother. Pharmacol.*, **9**, 140-147.

CALVERT AH, NEWELL DR, GUMBRELL LA, O'REILLY S, BURNELL M, BOXALL FE, SIDDIK ZH, JUDSON IR, GORE ME AND WILTSHAW E. (1989). Carboplatin dosage: prospective evaluation of a simple formula based on renal function. *J. Clin. Oncol.*, **7**, 1748-1756.

CAVALETTI G, MARZORATI L, BOGLIUN G, COLOMBO N, MARZOLA M, PITTELLI MR AND TREDICI G. (1992). Cisplatin-induced peripheral neurotoxicity is dependent on total dose-intensity and single dose-intensity. *Cancer*, **69**, 203-207.

CHILDS WJ, NICHOLLS EJ AND HORWICH A. (1992). The optimization of carboplatin dose in carboplatin, etoposide and bleomycin combination chemotherapy for good prognosis in metastatic nonseminomatous germ cell tumours of the testis. *Ann. Oncol.*, **3**, 291-296.

DAUGAARD G AND ABILDGAARD U. (1989). Cisplatin nephrotoxicity. A review. *Cancer Chemother. Pharmacol.*, **25**, 1-9.

EASTMAN A. (1986). Reevaluation of interaction of *cis*-dichloro(ethylenediammine)-platinum(II) with DNA. *Biochemistry*, **25**, 3912-3915.

EASTMAN A AND SCHULTE N. (1988). Enhanced DNA repair as a mechanism of resistance to *cis*-diamminedichloroplatinum(II). *Biochemistry*, **27**, 4730-4734.

EGORIN MJ, VAN ECHO DA, TIPPING SJ, OLMAN EA, WHITACRE MY, THOMPSON BW AND AISNER J. (1984). Pharmacokinetics and dosage reduction of *cis*-diammine(1,1-cyclobutanedicarboxylato)platinum(II) in patients with impaired renal function. *Cancer Res.*, **44**, 5432-5438.

EGORIN MJ, VAN ECHO DA, OLMAN EA, WHITACRE MY, FORREST A AND AISNER J. (1985). Prospective validation of a pharmacologically based dosing scheme for the *cis*-diamminedichloroplatinum (II) analogue diamminecyclobutanedicarboxylatoplatinum. *Cancer Res.*, **45**, 6502-6506.

FICHTINGER-SCHEPMAN AMJ, VAN OOSTEROM AT, LOHMAN PHM AND BERENDS F. (1987). *cis*-Diamminedichloroplatinum (II)-induced DNA adducts in peripheral leucocytes from seven cancer patients: quantitative immunochemical detection of the adduct induction and removal after a single dose of *cis*-Diamminedichloroplatinum (II). *Cancer Res.*, **47**, 3000-3004.

GERRITSEN VAN DER HOOP R, VECHT CHJ, VAN DER BURG MEL, ELDERSON A, BOOGERD W, HEIMANS JJ, VRIES EP, HOUWELINGEN VAN JC, JENNEKENS FHI, GISPEN WH AND NEIJT JP. (1990). Prevention of cisplatin neurotoxicity with an ACTH(4-9) analogue in patients with ovarian cancer. *N. Engl. J. Med.*, **322**, 89-94.

GILL I, MUGGIA FM, TERHEGGEN PMAB, MICHAEL C, PARKER RJ, KORTES V, GRUNBERG S, CHRISTIAN MC, REED E AND DEN ENGELSE L. (1991). Dose-escalation study of carboplatin (day 1) and cisplatin (day 3): tolerance and relation to leucocyte and buccal cell platinum-DNA adducts. *Ann. Oncol.*, **2**, 115-121.

GLOVER D, GLICK JH, WEILER C, FOX K AND GUERRY D. (1987). WR-2721 and high-dose cisplatin: an active combination in the treatment of metastatic melanoma. *J. Clin. Oncol.*, **5**, 574-578.

GOLDBERG JM AND LINDBLOM U. (1979). Standardized method of determining vibratory perception thresholds for diagnosis and screening in neurological investigation. *J. Neurol. Neurosurg. Psychiatry*, **42**, 793-803.

HAINSWORTH JD AND GRECO FA. (1993). Treatment of patients with cancer of an unknown primary site. *N. Engl. J. Med.*, **329**, 257-263.

HANSEN HH. (1992). Management of small-cell cancer of the lung. *Lancet*, **339**, 846-849.

HENGSTLER JG, FUCHS J AND OESCH F. (1992). DNA strand breaks and DNA cross-links in peripheral mononuclear blood cells of ovarian cancer patients during chemotherapy with cyclophosphamide/carboplatin. *Cancer Res.*, **52**, 5622-5626.

HIMMELSTEIN KJ, PATTON TF, BELT RJ, TAYLOR S, REPTA AJ AND STERNSON LA. (1981). Clinical kinetics of intact cisplatin and some related species. *Clin. Pharm. Ther.*, **29**, 658-664.

HOSMER DW AND LEMESHOW S. (1980). Goodness-of-fit tests for the multiple logistic regression model. *Communications Stat.*, **A9**, 1043-1069.

HOSMER DW AND LEMESHOW S. (1989). *Applied Logistic Regression*. Wiley: New York.

JODRELL DI, EGORIN MJ, CANETTA RM, LANGENBERG P, GOLDBLOOM EP, BURROUGHS JN, GOODLOW JL, TAN S AND WILTSHAW E. (1992). Relationships between carboplatin exposure and tumour response and toxicity in patients with ovarian cancer. *J. Clin. Oncol.*, **10**, 520-528.

KAYE SB, LEWIS CR, PAUL J, DUNCAN ID, GORDON HK, KITCHENER HC, CRUICKSHANK DJ, ATKINSON RJ, SOUKOP M, RANKIN EM, CASSIDY J, DAVIS JA, REED NS, CRAWFORD SM, MACLEAN A, SWAPP GA, SARKAR TK, KENNEDY JH AND SYMONDS RP. (1992). Randomized study of two doses of cisplatin with cyclophosphamide in epithelial ovarian cancer. *Lancet*, **340**, 329-333.

KRARUP-HANSEN A AND HANSEN HH. (1991). Chemotherapy in malignant mesothelioma: a review. *Cancer Chemother. Pharmacol.*, **28**, 319-330.

LEVIN L AND HRYNIUK WM. (1987). Dose-intensity analysis of chemotherapy regimens in ovarian carcinoma. *J. Clin. Oncol.*, **5**, 756-767.

LEVIN L, SIMON R AND HRYNIUK W. (1993). Importance of multiagent chemotherapy regimens in ovarian carcinoma: Dose-intensity analysis. *J. Natl Cancer Inst.*, **85**, 1732-1742.

LOEHRER PJ AND EINHORN LH. (1984). Diagnosis and treatment. Drugs five years later. Cisplatin. *Ann. Intern. Med.*, **100**, 704-713.

LOGOTHETIS CJ AND AMATO RJ. (1992). Dose-intensity in germ cell tumours: Lessons learned? *J. Natl Cancer Inst.*, **84**, 1686-1687.

MA J, VERWEIJ J, PLANTING AST, DE BOER-DENNERT M, INGEN VAN HE, VAN DER BURG MEL, STOTER G AND SCHELLENS JHM. (1995). Current sample handling methods for measurement of platinum-DNA-adducts in leucocytes in man lead to discrepant results in DNA-adduct levels and DNA-repair. *Br. J. Cancer*, **71**, 512-517.

MARKMAN M. (1993). The role of platinum dose-intensity in the management of ovarian cancer. *J. Cancer Res. Clin. Oncol.*, **119**, 511-512.

MOTZER RJ, BOSL GJ, GELLER NL, PENENBERG D, YAGODA A, GOLBEY R, WHITMORE WF, FAIR WR, SOGANI P, HERR H, MORSE M, CAREY RW AND VOGELZANG N. (1988). Advanced seminoma: the role of chemotherapy and adjunctive surgery. *Ann. Intern Med.*, **108**, 513-518.

MOTZER RJ, REED E, PERERA F, TANG D, SHAMKHANI H, POIRIER MC, TSA W-Y, PARKER RJ AND BOSL GJ. (1994). Platinum-DNA adducts assayed in leucocytes of patients with germ cell tumours measured by atomic absorbance spectrometry and enzyme-linked immunosorbent assay. *Cancer*, **73**, 2843-2852.

NATIONAL CANCER INSTITUTE. (1988). *Common Toxicity Criteria*. Cancer Therapy Evaluation Program, Division of Cancer Treatment, National Cancer Institute: Bethesda, MD.

OZOLS RF. (1989). Cisplatin dose-intensity. *Semin. Oncol.*, **16**, 22-30.

OZOLS RF, IDHE DC, LINEHAN M, JACOB J, OSTCHEGA Y AND YOUNG RC. (1988). A randomized trial of standard chemotherapy *vs* a high-dose chemotherapy regimen in the treatment of poor prognosis nonseminomatous germ-cell tumours. *J. Clin. Oncol.*, **6**, 1031-1040.

PACCAGNELLA A, ORLANDO A, MARCHIORI C, ZORAT PL, CAVANIGLIA G, SILENI VC, JIRILLO A, TOMIO L, FILA G, FEDE A, ENDRIZZI L, BARI M, SAMPOGNARO E, BALLI M, GAVA A, PAPPAGALLO GL AND FIORENTINO MV. (1994). Phase II trial of initial chemotherapy in stage III or IV head and neck cancers: a study by the gruppo di studio sui tumori della testa e del collo. *J. Natl Cancer Inst.*, **86**, 265-272.

PARKER RJ, GILL I, TARONE R, VIONNET JA, GRUNBERG S, MUGGIA FM AND REED E. (1991). Platinum-DNA damage in leucocyte DNA of patients receiving carboplatin and cisplatin chemotherapy, measured by atomic absorption spectrometry. *Carcinogenesis*, **12**, 1253-1258.

PLANTING AST, DE BOER M, VAN DER BURG MEL, STOTER G AND VERWEIJ J. (1991). Pilot study of a short course of weekly high dose cisplatin combined with oral etoposide. *Eur. J. Cancer*, 27 (suppl. 2),1203.

PLANTING AST, STOTER G AND VERWEIJ J. (1992). Phase II study of a short course of weekly cisplatin in locally advanced and recurrent squamous cell carcinoma of the head and neck. *Proc. Am. Assoc. Cancer Res.*, **33**, 225.

PLANTING AST, VAN DER BURG MEL, DE BOER-DENNERT M, STOTER G AND VERWEIJ J. (1993*a*). Phase I/II study of a short course of weekly cisplatin in patients with advanced solid tumours. *Br. J. Cancer*, **68**, 789-792.

PLANTING AST, GOEY S, DE BOER-DENNERT M, VECHT CH, STOTER G AND VERWEIJ J. (1993*b*). Phase II study of a short course of weekly cisplatin combined with oral etoposide in pleural mesothelioma. *Eur. J. Cancer*, **29A**, S184.

PLANTING AST, SCHELLENS JHM, GOEY SH, VAN DER BURG MEL, DE BOER-DENNERT M, STOTER G AND VERWEIJ J. (1994). Weekly high-dose cisplatin in malignant pleural mesothelioma. *Ann. Oncol.*, **5**, 373–374.

POIRIER MC, REED E, LITTERST CL, KATZ D AND GUPTA-BURT G. (1992). Persistence of platinum-ammine-DNA adducts in gonads and kidneys of rats and multiple tissues from cancer patients. *Cancer Res.*, **52**, 149–153.

POIRIER MC, REED E, SHAMKHANI H, TARONE RE AND GUPTA-BURT S. (1993). Platinum drug-DNA interactions in human tissue measured by cisplatin-DNA enzyme-linked immunosorbent assay and atomic absorbance spectroscopy. *Environm. Health Persp.*, **99**, 149–154.

REECE PHA, STAFFORD I, RUSSELL J, KHAN M AND FILL PG. (1987). Creatinine clearance as a predictor of ultrafilterable platinum disposition in cancer patients treated with cisplatin: relationship between peak ultrafilterable platinum plasma levels and nephrotoxicity. *J. Clin. Oncol.*, **5**, 304–309.

REECE PA, STAFFORD I, ABBOTT RL, ANDERSON C, DENHAM J, FREEMAN S, MORRIS RG, GILL PG AND OLWENY CL. (1989). Two- *versus* 24-hour infusion of cisplatin: pharmacokinetic considerations. *J. Clin. Oncol.*, **7**, P270–275.

REED E, YUSPA SH, ZWELLING LA, OZOLS RF AND POIRIER MC. (1986). Quantitation of *cis*-diamminedichloroplatinum II (cisplatin)-DNA-intrastrand adducts in testicular and ovarian cancer patients receiving cisplatin chemotherapy. *J. Clin. Invest.*, **77**, 545–550.

REED E, OZOLS RF, TARONE R, YUSPA SH AND POIRIER MC. (1987). Platinum-DNA adducts in leucocyte DNA correlate with disease response in ovarian cancer patients receiving platinum-based chemotherapy. *Proc. Natl Acad. Sci. USA*, **84**, 5024–5028.

REED E, OZOLS RF, TARONE R, YUSPA SH AND POIRIER MC. (1988*a*). The measurements of cisDDP-DNA adduct levels in testicular cancer patients. *Carcinogenesis*, **9**, 1909–1911.

REED E, SAUERHOFF S AND POIRIER MC. (1988*b*). Quantitation of platinum-DNA binding after therapeutic levels of drug exposure – a novel use of graphite furnace spectrometry. *Atomic Spectroscopy*, **9**, 93–95.

REED E, OSTCHEGA Y, STEINBERG SM, YUSPA SH, YOUNG RC, OZOLS RF AND POIRIER MC. (1990). Evaluation of platinum-DNA adduct levels relative to known prognostic variables in a cohort of ovarian cancer patients. *Cancer Res.*, **50**, 2256–2260.

REED E, PARKER RJ, GILL I, BICHER A, DABHOLKAR M, VIONNET JA, BOSTICK-BRUTON F, TARONE R AND MUGGIA FM. (1993). Platinum-DNA adduct in leucocyte DNA of a cohort of 49 patients with 24 different types of malignancies. *Cancer Res.*, **53**, 3694–3699.

ROELOFS RI, HRUSHESKY W, ROGIN J AND ROSENBERG L. (1984). Peripheral sensory neuropathy and cisplatin chemotherapy. *Neurology*, **34**, 934–938.

ROTH JA, FOSSELLA F, KOMAKI R, RYAN B, PUTNAM JB, LEE JS, DHINGRA H, DECARO L, CHASEN M, MCGAVRAN M, ATKINSON EN AND HONG WK. (1994). A randomized trial comparing perioperative chemotherapy and surgery with surgery alone in resectable stage IIIA non-small-cell lung cancer. *J. Natl Cancer Inst.*, **86**, 673–680.

SAMSON MK, RIVKIN SE, JONES SE, COSTANZI JJ, LOBUGLIO AF, STEPHENS RL, GEHAN EA AND CUMMINGS AD. (1984). Dose–response and dose–survival advantage for high *versus* low-dose cisplatin combined with vinblastine and bleomycin in disseminated testicular cancer. *Cancer*, **53**, 1029–1035.

SHEINER LB AND BEAL SL. (1985). Pharmacokinetic parameter estimates from several least squares procedures: superiority of extended least squares. *J. Pharmacokinet. Biopharmaceut.*, **13**, 185–210.

SIEGAL T AND HAIM N. (1990). Cisplatin-induced peripheral neuropathy. *Cancer*, **66**, 1117–1123.

SLOTMAN GJ, DOOLITTLE CHH AND GLICKSMAN AS. (1992). Preoperative combined chemotherapy and radiation therapy plus radical surgery in advanced head and neck cancer. *Cancer*, **69**, 2736–2743.

STOTER G, SPLINTER TAW, CHILD JA, FOSSA SD, DENIS L, VAN OOSTEROM AT, DE PAUW M AND SYLVESTER R. (1987). Combination chemotherapy with cisplatin and methotrexate in advanced transitional cell cancer of the bladder. *J. Urol.*, **137**, 663–667.

STOTER G, LOEHRER PJ AND LEVI J. (1996). Management of non-seminomatous germ cell tumours stage II and good risk stage III and IV. In *Principles and Practice of Genitourinary Oncology*. Raghavan D, Scher HJ, Leibel SA (eds) JB Lippincott, In press.

WORLD HEALTH ORGANIZATION. (1979). *Handbook for Reporting Results of Cancer Treatment*. World Health Organization: Geneva.